# The cholesterol esterification inhibitor avasimibe suppresses tumour proliferation and metastasis via the E2F-1 signalling pathway in prostate cancer

**DOI:** 10.1186/s12935-021-02175-5

**Published:** 2021-08-30

**Authors:** Kangping Xiong, Gang Wang, Tianchen Peng, Fenfang Zhou, Siming Chen, Wei Liu, Lingao Ju, Yu Xiao, Kaiyu Qian, Xinghuan Wang

**Affiliations:** 1 Department of Urology, Zhongnan Hospital of Wuhan University, Wuhan, China; 2 Department of Biological Repositories, Zhongnan Hospital of Wuhan University, Wuhan, China; 3 Human Genetic Resource Preservation Center of Hubei Province, Wuhan, China; 4 Human Genetic Resource Preservation Center of Wuhan University, Wuhan, China; 5 Laboratory of Precision Medicine, Zhongnan Hospital of Wuhan University, Wuhan, China; 6 Wuhan Research Center for Infectious Diseases and Cancer, Chinese Academy of Medical Sciences, Wuhan, China; 7 Medical Research Institute, Wuhan University, Wuhan, China

**Keywords:** Prostate cancer, Avasimibe, E2F-1, Cell cycle, Metastasis

## Abstract

**Background:**

New effective drugs for prostate cancer (PCa) treatment are urgently needed. Avasimibe was recently identified as a promising drug for anticancer therapies. The main purpose of this study was to explore the effects and the underlying mechanisms of avasimibe in prostate cancer.

**Methods:**

In this study, MTT and clonogenic survival assays were performed to detect cell proliferation after avasimibe treatment. The effect of avasimibe on cell migration was measured by wound healing and transwell migration assays. Cell cycle distribution and apoptosis were detected by flow cytometry. Immunofluorescence staining and western blot analysis were used to detect the expression of cell cycle-related proteins and epithelial-mesenchymal transition (EMT)-related proteins. In vivo, the antitumour effects of avasimibe were evaluated using a xenograft model and pulmonary metastasis model.

**Results:**

The study found that avasimibe suppresses tumour growth and triggers G1 phase arrest. Moreover, the expression of the cell cycle-related proteins CDK2/4/6, Cyclin D1 and Cyclin A1 + A2 was significantly increased and p21 expression was decreased after avasimibe treatment. The migration of PCa cells was attenuated after treatment with avasimibe, followed by the downregulation of the expression of the EMT-related proteins N-cadherin, β-catenin, vimentin, Snail and MMP9 and upregulation of E-cadherin expression. Moreover, E2F-1 was elevated after treatment with avasimibe. After knockdown of E2F-1 expression, the inhibition of cell proliferation and migration caused by avasimibe was significantly recovered. The results of the xenograft model showed that avasimibe suppressed tumour growth in vivo. Immunofluorescence staining revealed lower levels of Ki67 and higher levels of E2F-1 in tumour tissues of the avasimibe group than those of the control group. A pulmonary metastasis model also confirmed the inhibition of PCa metastasis by avasimibe. The number of lung metastatic foci in the avasimibe group was significantly decreased compared with that in the control group.

**Conclusions:**

Our results suggest that avasimibe can suppress tumour proliferation and metastasis via the E2F-1 signalling pathway. These findings demonstrate the potential of avasimibe as a new effective drug for PCa treatment.

**Supplementary Information:**

The online version contains supplementary material available at 10.1186/s12935-021-02175-5.

## Background

Prostate cancer (PCa), a common urinary malignancy, is the second most common cause of male morbidity and the fifth most common cause of male mortality worldwide [1]. PCa occupies the first place of morbidity, and its mortality rate ranks 2nd among malignant tumours of males in the United States [2]. PCa mostly occurs in the peripheral zone, without obvious symptoms in the early stage, and metastatic disease is the leading cause of death in prostate cancer patients [3]. The five-year survival rate of early PCa is 99%; once metastasis occurs, the five-year survival rate is reduced to 28% [4]. Surgical resection, androgen deprivation therapies and castration therapy are the usual methods for the treatment of early PCa [5]. However, the postoperative quality of life of the patient can be substantially affected after radical prostatectomy, and even after antiandrogenic therapy, the patient may develop lethal castration-resistant prostate cancer (CRPC). Once distant metastases develop, patients largely have a poor outcome even after intensive multimodal therapy [6]. Thus, more efficacious and less toxic drugs to treat PCa are needed.

Metabolic reprogramming is considered an important hallmark of cancer, and activated de novo lipid synthesis has been recognized as an emerging hallmark of cancers [7]. Along with lipogenesis, lipolysis has been found to be elevated in a range of cancer types [8]. Lipid droplets (LDs) play multiple crucial roles in this process. The synthesized lipids can be transported into LDs for storage; however, LDs can be hydrolysed to provide lipids [9]. It was also reported that cholesteryl ester (CE) accumulation in prostate cancer cells was due to the loss of phosphatase and tensin homolog (PTEN), which leads to PI3K/AKT pathway activation [9]. As an important lipid, cholesterol is a vital component of the cell membrane and is essential for a diverse range of cellular processes, such as hormone signalling and lipid metabolism [10]. However, cholesterol is not always beneficial, excess free cholesterol is highly cytotoxic, and the level of cholesterol must be controlled and regulated at multiple levels to maintain cell viability [11]. To maintain cholesterol homeostasis in the cell, cells excrete a portion of the excess free cholesterol, and the remaining free cholesterol is stored in LDs by synthetic CE. CE, an important component of LDs, was found to build up in large amounts in high-grade and metastatic human PCa tissues [9]. As an important buffer mechanism to control intracellular free cholesterol concentration, CE plays an essential role in the onset and progression of prostate cancer [9].

The key enzyme that mediates cholesterol synthesis of CE is sterol O-acyltransferase (SOAT), also referred to as acyl-coenzyme A: cholesterol acyltransferase [12]. Avasimibe, a key drug that targets SOAT to deplete CE, has been tested in clinical trials to treat atherosclerosis and has shown good biocompatibility and good tolerance [13]. Moreover, this drug has been verified to potently inhibit proliferation or metastasis in multiple types of cancers [9, 14, 15]. A study reported that avasimibe could inhibit the expression of LINC00339 to suppress the proliferation and metastasis of glioma cells [15]. Avasimibe could also suppress PCa cell proliferation by downregulating the expression of low-density lipoprotein receptors and decreasing low-density lipoprotein uptake [9]; this is also one of the pathways through which the E2F-1 protein exerts its anti-cancer effects. As a transcription factor, E2F-1 is functionally complex and powerful. In addition to controlling downstream gene transcription to promote tumour cell apoptosis and thus suppress cancer progression, E2F-1 is also actively involved in lipid metabolism in cells, and low-density lipoprotein-cholesterol can promote the expression of E2F-1 in endothelial cells [16]. Some studies have revealed that E2F-1 depletion can promote the expression of low-density lipoprotein receptors and thus influence the development of tumours by affecting lipid uptake by tumour cells [17]. Given the abnormal lipid accumulation in prostate cancer tissue and the unique function of avasimibe, we speculate that avasimibe exerts its anti-cancer effect by regulating E2F-1. However, the exact mechanism of avasimibe’s effect on prostate cancer remains to be further explored.

In this study, we found that the cholesterol esterification inhibitor avasimibe can suppress tumour proliferation and metastasis in vitro and in vivo accompanied by upregulation of E2F-1 protein expression. Knockdown of E2F-1 expression could partly rescue the inhibition of proliferation and migration in avasimibe-treated PCa cells. The abovementioned results confirmed that avasimibe could suppress the proliferation and migration of PCa cells via the E2F-1 signalling pathway.

## Materials and methods

### Cell culture and reagents/chemicals

PCa cells (PC-3, DU 145) were kindly provided by Cell Bank, Chinese Academy of Sciences and were authenticated by STR profiling. No mycoplasm contamination. PC-3 cells were cultured in 1640 medium (RPMI 1640) and completed with 10% FBS (Gibco, Australia). DU 145 cells were maintained in DMEM completed with 10% FBS (Gibco, Australia). PCa cells were cultured at 37 °C and 5% CO_2_ in a cell incubator.

Avasimibe (MedChemExpress, China) was dissolved in DMSO (stock solution of 100 mM) for use in cell culture treatments according to the instructions. The essential reagents/chemicals used in the experiment are listed in Additional file 1: Table S1.

### Cell transfection and construction of stable cell lines

E2F-1 siRNA and control siRNA were purchased from Gene Pharma Gene Co Ltd. The sequence of E2F-1 siRNA-1 was 5′-GCGCAUCUAUGACAUCACCTT-3′, E2F-1 siRNA-2 was 5′-GGACUCUUCGGAGAACUUUTT-3′, and control siRNA was 5′-UUCUCCGAACGUGUCACGUTT-3′. PCa cells were transfected using Lipofectamine 3000. qRT-PCR and western blotting were carried out to verify the alterations in mRNA and protein expression after 48 h of transfection. Transfection was carried out according to the previous work [18].

PC-3-GFP vector virus was obtained from Gene Pharma. For selection of stable cell lines, PC-3 cells were transfected with control shRNA for 48 h, and 2.5 µg/ml puromycin was added to the medium at 2 weeks for further selection.

### MTT assay

Briefly, PCa cells were plated in 96-well plates (3000 cells/well; 200 µl of medium) for 1 day and treated with avasimibe (0, 0.25, 5, 10, 20, 40 and 80 µM) for 1, 2, and 3 days. The cells were incubated with 20 µl of MTT (5 mg/ml/well) for 4 h at 37 °C. After discarding the supernatant, the MTT formazan crystals were dissolved in 200 µl/well DMSO, and a microplate reader was applied to measure the OD values at 490 nm [19, 20].

### Clonogenic survival assay

PCa cells were placed onto a six-well plate (1500 cells per well). After 1 day, the normal medium was replaced with avasimibe working solution. The cells were cultured for 10 to 15 days until they grew into colonies. The medium was discarded, then, the cells were fixed for 1 h with 4% paraformaldehyde (PFA) and stained for half an hour in 0.1% crystal violet. The colonies were counted using Image-Pro Plus.

### Wound healing assay

Avasimibe-treated PCa cells were grown in six-well plates until the cells reached 95% confluence. Then, the cell monolayer was scratched with a 1-ml sterile blue micropipette tips. After the cells were washed twice with PBS, they were cultured in medium supplemented with 2% FBS and different concentrations of avasimibe for 12 h. Then, the cells were photographed with an inverted fluorescence microscope at premarked points with white light at 0 and 12 h. The horizontal distance between the edges of the scratch was measured by Photoshop. Migration rate = 1 − (12 h scratch distance/0 h initial distance).

### Transwell migration assay

Cell migration was evaluated by a Transwell chamber system. The cells were pretreated with the avasimibe in a six-well plate for 48 h, then avasimibe-treated PCa cells (1.2 × 10^5^ PC3 or 8 × 10^4^ DU 145 cells) in 200 µl of medium (serum-free) were added to the top transwell chamber, and 600 µl of normal culture medium was placed in the lower chamber. After incubation for 1 day, the chambers were fixed with 4% PFA for half an hour and stained in 0.1% crystal violet for 1 h. The chambers were photographed and assessed by an inverted phase contrast microscope in five random fields.

### Flow cytometry for cell cycle analysis

After transfection for 1 day, cells were treated with avasimibe working solution for 1 day. Avasimibe-treated PCa cells were collected and then washed with PBS three times. A total of 1 × 10^6^ cells were harvested for cell cycle staining, and then, 500 µl of 1× DNA Staining Solution and 5 µl of permeabilization solution were placed in tubes in the dark for staining for 30 min. 1 × 10^4^ cells of the sample were assessed by flow cytometry (Cytoflex, Beckman, China) as described before [21]. Data were analysed with FlowJo Software.

### Flow cytometry for apoptosis

The steps of the apoptosis assay were carried out as described before [21]. 100 µl of binding buffer (1×) was used to resuspend PCa cells, and the avasimibe-treated PCa cells were stained with FITC-annexin V (5 µl) and propidium iodide (PI, 5 µl) for 10 min in dark conditions at 25 °C. Then, 1× binding buffer was added to the mixture to bring the total volume to 500 µl, and the samples were assessed by flow cytometry. Percentage of apoptosis = percentage of late apoptosis + percentage of early apoptosis.

### Flow cytometry for reactive oxygen species (ROS)

Flow cytometric analysis was performed to assess the intracellular levels of ROS. Avasimibe-treated PCa cells were stained with 2′,7′-Dichlorodihydrofluorescein diacetate (DCFH-DA,10 µM) for 20 min at 25 °C, protected from light, and then washed with PBS. The ROS level was assessed by flow cytometry.

### qRT-PCR

Total RNA was extracted by a commercial HiPure Total RNA Mini Kit. The experiment was performed according to the manufacturer’s protocols. We measured the RNA concentration using a NanoDrop® ND-2000 UV-Vis spectrophotometer. RNA (1 µg) was reverse transcribed into cDNA using the ReverTrace qPCR RT Kit. All the primer sequences used in the experiment are listed in Additional file 1: Table S2. Five hundred nanograms of cDNA were used for qRT-PCR in a volume of 20 µl. The qRT-PCR cycling conditions were as follows: 95 °C for 10 min, followed by 40 cycles of 95 °C (denaturing) for 15 s and 60 °C (annealing and extension) for 60 s. The major steps were previously described [22].

### Western blot analysis

PCa cells were lysed on ice with lysis buffer for 30 min, and then, the cell lysate was centrifuged at 13,000*g* for 15 min. Separation and detection of the proteins were carried out as described previously [21]. Primary and secondary antibodies are summarized in Additional file 1: Tables S3 and S4.

### Haematoxylin and eosin staining (H&E)

The tissue sections (5 μm) were serially deparaffinized and rehydrated by graded alcohol (100%, 100%, 95%, 80% and 70%) and H_2_O. Then, the sections were stained with 10% haematoxylin and stained with 1% eosin for 10 min (Sigma–Aldrich, USA). The tissue sections were scanned with an inverted phase contrast microscope (Leica, Germany).

### Immunofluorescence staining

Samples (cells or tissue sections) were fixed in 4% PFA for half an hour, washed with PBS then incubated with buffer (2 % BSA and 0.5% Triton X-100) for 1 h at 25 °C. Next, the cells were incubated with the corresponding primary antibody (Additional file 1: Table S3) for 2 h. In addition, the cells or tissue sections were washed with PBS three times and then incubated with secondary antibody (Additional file 1: Table S4) for 2 h. After that, the cells and tissue sections were washed with PBS. Finally, cells or tissue sections were incubated with DAPI (1:1000) for 5 min to label the nuclei. Immunofluorescence staining was photographed on a confocal microscope (Nikon, Japan).

### Xenograft model and pulmonary metastasis model

SPF male mice (BALB/c-nude, 4 weeks old) were obtained from River Laboratory Animal Technology Company. The mice were acclimated to the environment of the animal facility for seven days.

Tumour-bearing mice were constructed by inoculating 2 × 10^6^ PC-3 cells into the flanks of mice (n = 7). Seven days later, avasimibe (30 mg/kg, dissolved in DMSO and diluted in PBS containing 1% Tween-80) [9] and solvent were intraperitoneally injected on alternate days for 7 weeks (the stock solution had a concentration of 25 mg/ml) [21]. The mice were anaesthetized by intraperitoneal injection of pentobarbital (50 mg/kg) before euthanasia. Tumour volume was measured with a Vernier scale every other day for 7 weeks, and tumour volume was calculated as follows: tumour volume (mm^3^) = tumour length × width^2^/2. We separated the tumour tissues, and then, the tumour tissues were fixed in 4 % PFA and verified by H&E and immunofluorescence staining.

Pulmonary metastasis models were constructed by injecting 2 × 10^6^ PC-3 (LV-NC GFP-expressing) cells into the tail vein of mice (n = 5) [22]. Avasimibe (30 mg/kg) and solvent were administered as described above for 7 weeks. The fluorescence intensity of lung metastasis tumours was measured using a Fusion FX7 Spectra Imaging system. Then, the lungs were surgically exposed and collected for further analysis by H&E.

The animal experiment was performed in accordance with the Declaration of Helsinki and complied with the “Guidance Suggestions on the Care and Use of Animals” of China.

### Gene set enrichment analysis (GSEA)

Raw data were obtained from The Cancer Genome Atlas. First, we standardized the data to eliminate the offset and ensure the homogeneity and integrity of the data. Then, to form the expression matrix, we used the “limma” R package to make comparisons between PCa and normal prostate tissues to screen the differentially expressed genes. A total of 499 PCa tissue samples were divided into two groups by taking the median value of SOAT1. Finally, we performed GSEA on SOAT1. We selected c2.cp.kegg.v7.3.symbols.gmt as the reference sets to annotate all gene sets.

### Statistics

All experiments were repeated three or more times, and the representative data were from three independent experiments. The statistical significance of the differences was calculated with two-tailed Student’s t test or one-way ANOVA. Statistical significance are as follows: *n.s.* = not statistically significant, *p < 0.05, ** p < 0.01, *** p < 0.001.

## Results

### Avasimibe reduced proliferation in the PCa cells

To determine the biological function of SOAT1 in PCa progression, we performed GSEA using 499 PCa samples. GSEA showed that SOAT1 expression was positively correlated with fatty acid metabolism (Additional file 1: Fig. S1a) and negatively correlated with oxidative phosphorylation (Additional file 1: Fig. S1b). In addition, PCa was significantly enriched (Additional file 1: Fig. S1c). The GEPIA database confirmed that SOAT1 was overexpressed in PCa samples (Additional file 1: Fig. S1e).

**Fig. 1:**
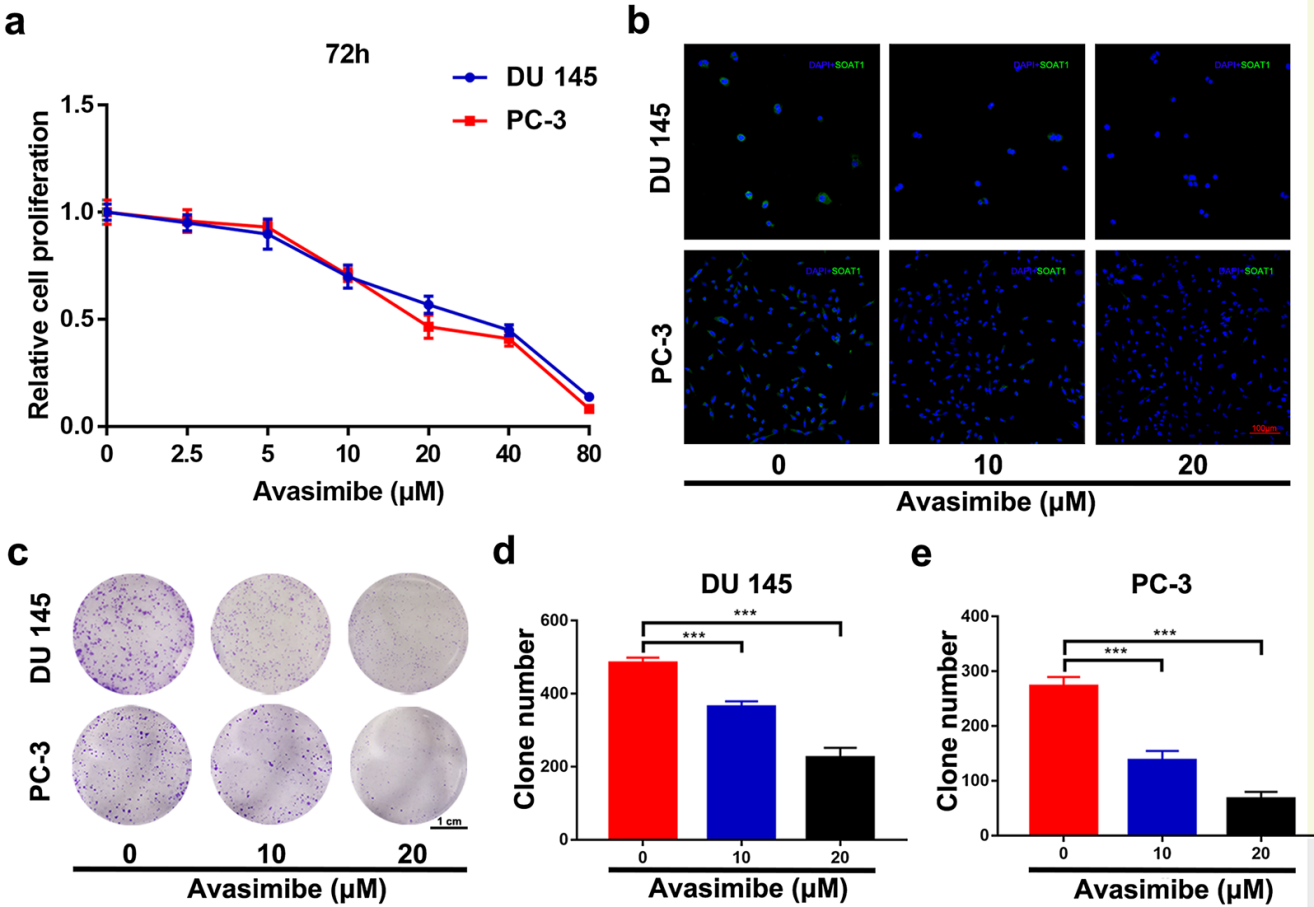
Avasimibe inhibited proliferation in PCa cells.** a** MTT assays were used to evaluate cell growth after treatment with various concentrations of avasimibe (0, 2.5, 5, 10, 20, 40, 80 µM) for 72 h; **b** immunofluorescence staining of SOAT1 to confirm the effect of avasimibe; the scale bar is 100 μm; **c** clonogenic survival assays were used to detect the proliferation of the avasimibe-treated PCa cell lines (PC-3, DU 145); the scale bar is 1 cm; **d** statistical analysis of the clone number of DU 145 cells; **e** statistical analysis of the clone number of PC-3 cells. Representative images are from three individual experiments. ***p < 0.001

To investigate the effect of avasimibe on cell viability of PCa cells (PC-3 and DU 145), we treated PCa cells with avasimibe at different concentrations (0, 2.5, 5, 10, 20, 40 and 80 µM) for 24 h (Additional file 1: Fig. S2a), 48 h (Additional file 1: Fig. S2b) and 72 h (Fig. 1a). Cell viability was measured by MTT assays, and we found that avasimibe dose dependently inhibited PC-3 and DU 145 cell viability. Immunofluorescence staining showed a significant reduction in SOAT1 expression (Fig. 1b) in the avasimibe-treated PCa cells. Clonogenic survival assays (Fig. 1c) and statistical analysis (Fig. 1d-e) confirmed that avasimibe could inhibit the colony formation of PCa cells.

### Avasimibe induced G1 phase cell cycle arrest of PCa cells

**Fig. 2:**
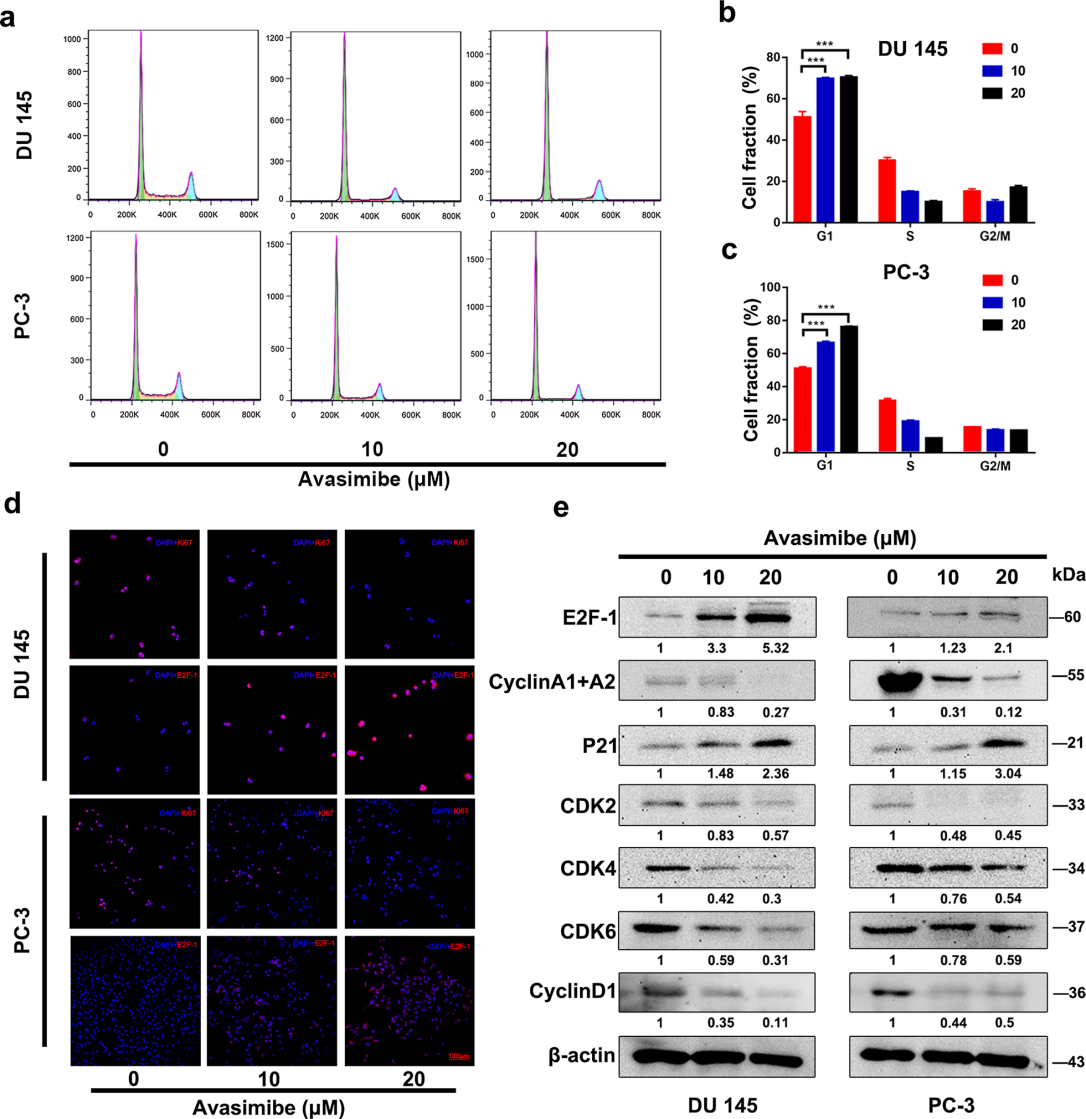
Avasimibe induced G1 phase cycle arrest and altered the G1 phase-related protein levels in PCa cells.** a** Flow cytometric analysis to detect the cell cycle for the 48 h avasimibe-treated PCa cell lines; **b** quantitative results of DU 145 cell fractions; **c** quantitative results of PC-3 cell fractions; **d** immunofluorescence staining of E2F-1 and Ki67 in PCa cells (DU 145 and PC-3) after avasimibe treatment; **e** protein levels of G1 phase-related proteins (CDK2/4/6, Cyclin D1/A1 + A2, p21 and E2F-1) in 48 h avasimibe-treated PCa cells were estimated by Western blotting. **p < 0.01, ***p < 0.001, the scale bar is 100 μm

To explore the cause of the decreased proliferation, we analysed the cell cycle by flow cytometry in PCa cells. The results indicated that avasimibe treatment for 48 h could trigger G1 phase arrest in a dose-dependent fashion (Fig. 2a–c). Western blotting was used to analyse the G1 phase-related protein levels. The results revealed decreased levels of CDK2, CDK4, CDK6, Cyclin D1, and Cyclin A1 + A2 and increased levels of p21 in the avasimibe group (Fig. 2e). Moreover, the E2F-1 protein level increased after avasimibe treatment, and immunofluorescence staining also confirmed a significant increase in E2F-1 expression and a decrease in Ki67 expression (Fig. 2d).

In addition, to further determine whether avasimibe-induced inhibition of proliferation was attributable to apoptosis, we used flow cytometry to detect the percentage of apoptosis. Compared with that of the control group, the percentage of apoptosis was not significantly different even in the high concentration group in PCa cells (Additional file 1: Fig. S3a, b).

### Avasimibe induced ROS accumulation in PCa cells

Multiple studies have reported that high cholesterol induces an increase in ROS levels [23, 24], and excessive ROS accumulation may irreversibly damage cells, which partially inhibits tumour growth and metastasis [25, 26]. Our results revealed that intracellular ROS levels were significantly increased in PCa cells after treatment with a high concentration of avasimibe (Additional file 1: Fig. S4a, b). Furthermore, the protein levels of Catalase and SOD2 were upregulated (Additional file 1: Fig. S4c).

### Avasimibe suppressed PCa cell migration and altered the levels of epithelial-mesenchymal transition (EMT)-related proteins

**Fig. 3:**
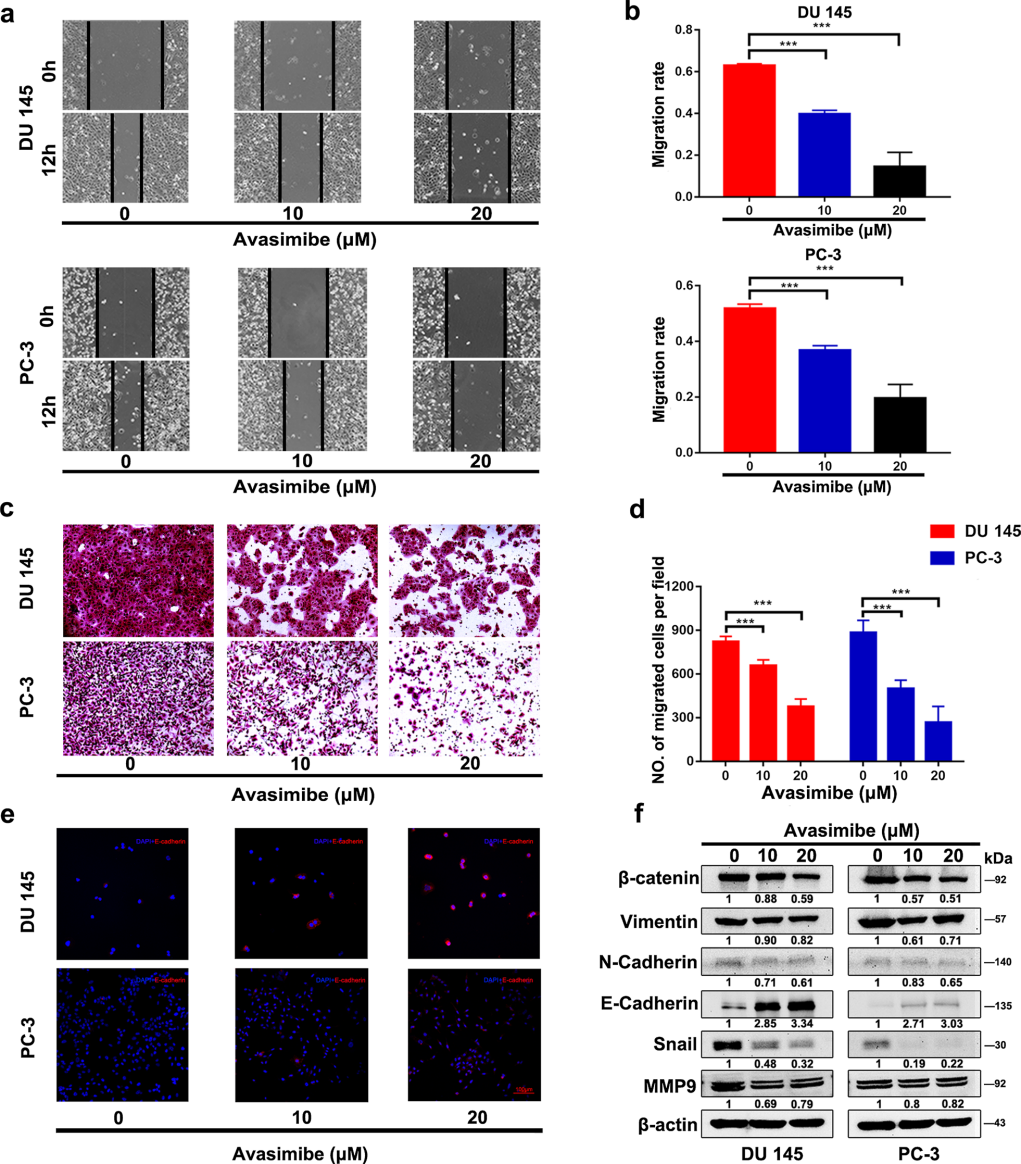
Avasimibe suppressed PCa cell migration.** a** Wound healing assays of PCa cells after 48 h of avasimibe treatment; **b** quantitative results of the migration rate; **c** Transwell assays of PCa cells after 48 h of avasimibe treatment; **d** the quantitative results of the Transwell assays; **e** immunofluorescence staining of E-cadherin in PCa cells after avasimibe treatment; **f** protein levels of EMT-related proteins (β-catenin, Vimentin, N-cadherin, Snail, MMP9 and E-cadherin) in 48 h-avasimibe-treated PCa cells were determined by Western blotting. ***p < 0.001

Wound healing (Fig. 3a, b) and transwell migration assays (Fig. 3c, d) were used to explore the effects of avasimibe on cell migration. The migration rate in the wound healing assays was calculated after 12 h of treatment with avasimibe. Compared with the vehicle group, treatment with low or high concentrations of avasimibe significantly decreased the migration rate of DU 145 and PC-3 cells (Fig. 3b). Avasimibe-inhibited PCa cell metastasis was also confirmed by transwell assays (Fig. 3d). GSEA results showed that samples with high expression were enriched in adherens junctions (Additional file 1: Fig. S1d). Adherens junctions are frequently altered during EMT. Avasimibe reduced the expression of β-catenin, Vimentin, N-cadherin, Snail and MMP9, which are tightly associated with EMT (Fig. 3f). Consistently, avasimibe upregulated the expression of E-cadherin in both PCa cell lines, which was further confirmed by western blots (Fig. 3f) and immunofluorescence staining (Fig. 3e).

### Avasimibe suppressed PCa cell growth and metastasis in vivo

**Fig. 4:**
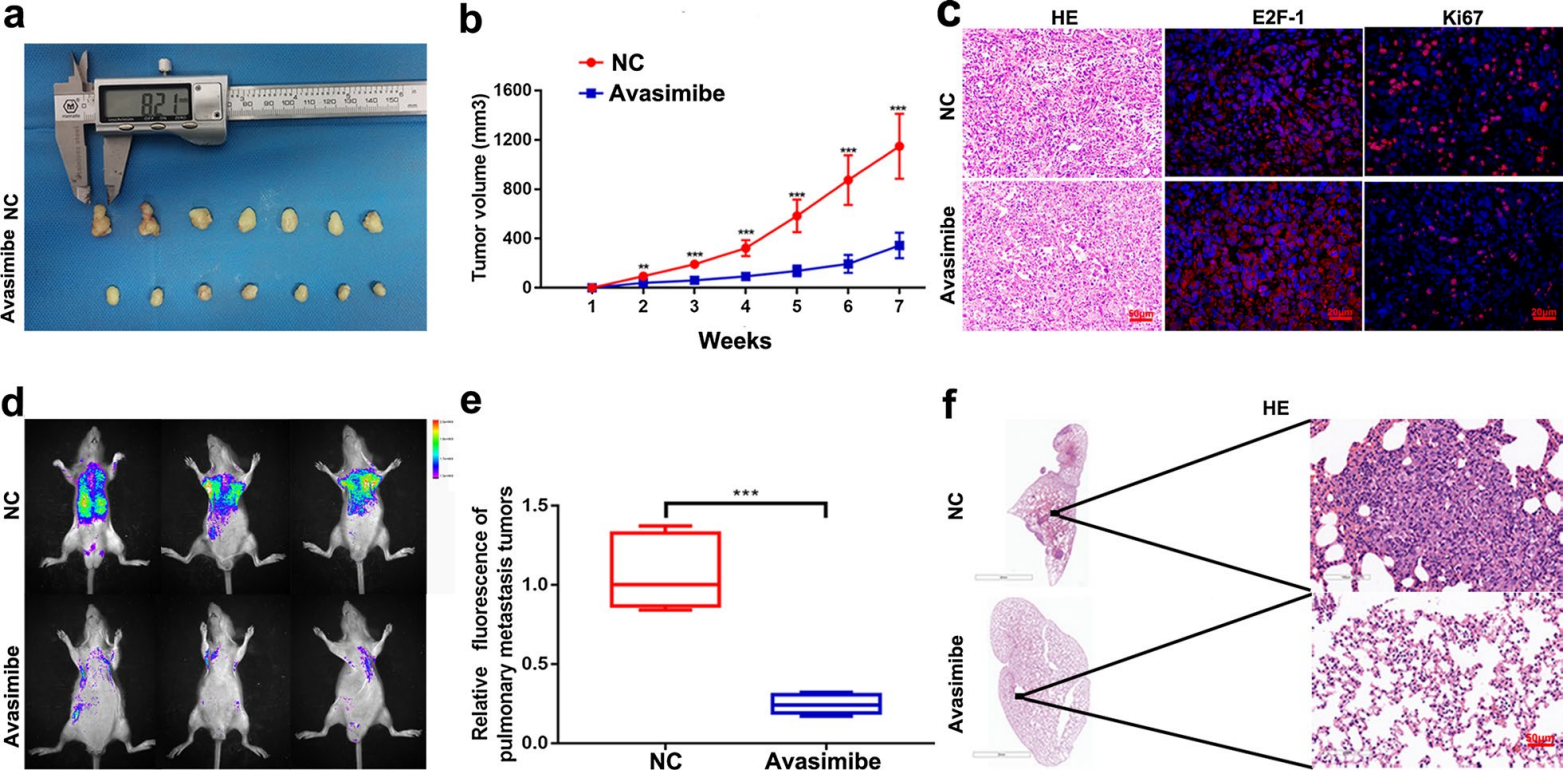
Avasimibe inhibited PCa growth and migration in vivo. **a** Tumour-bearing models were constructed by inoculating 2 × 10^6^ PC-3 cells into the flanks of mice (n = 7). Seven days later, avasimibe (30 mg/kg) and PBS were injected intraperitoneally on alternate days for 7 weeks. We separated and weighed the tumour tissues; **b** volume of the tumours; **c** H&E staining and immunofluorescence staining of tumours to assess the expression of Ki67 and E2F-1; **d** pulmonary metastasis models were constructed by injecting 2 × 10^6^ PC-3 cells (LV-NC GFP-expressing) into the tail vein of mice (n = 5). Avasimibe (30 mg/kg) and PBS were administered as described above for 7 weeks. **e** Fluorescence intensity of lung metastatic tumours was detected and statistically analysed. **f** H&E staining of the lungs. ***p < 0.001

A xenograft model was established by subcutaneously transplanting PC-3 cells, and our study found that avasimibe reduced tumour volume compared with that of the control group (Fig. 4a, b). The inhibitory effect of avasimibe on PCa cell growth was further confirmed by H&E staining and Ki67 immunofluorescence staining of xenograft tumours (Fig. 4c). An upregulation of E2F-1 expression was also observed in the avasimibe group by immunofluorescence staining (Fig. 4c).

We established a pulmonary metastasis model by intravenous tail vein injection of GFP-expressing PC-3 LV-NC cells, and the fluorescence intensity of the GFP-expressing PC-3 LV-NC cells was assessed to evaluate the migratory capacity. The fluorescence intensity of pulmonary metastatic tumours was weaker in the avasimibe group than the control group (Fig. 4d, e). H&E staining of lung tissues showed that avasimibe treatment could inhibit the number of pulmonary metastatic tumours (Fig. 4f).

### Avasimibe-induced inhibition of cell proliferation and migration was partially rescued by E2F-1 knockdown

**Fig. 5:**
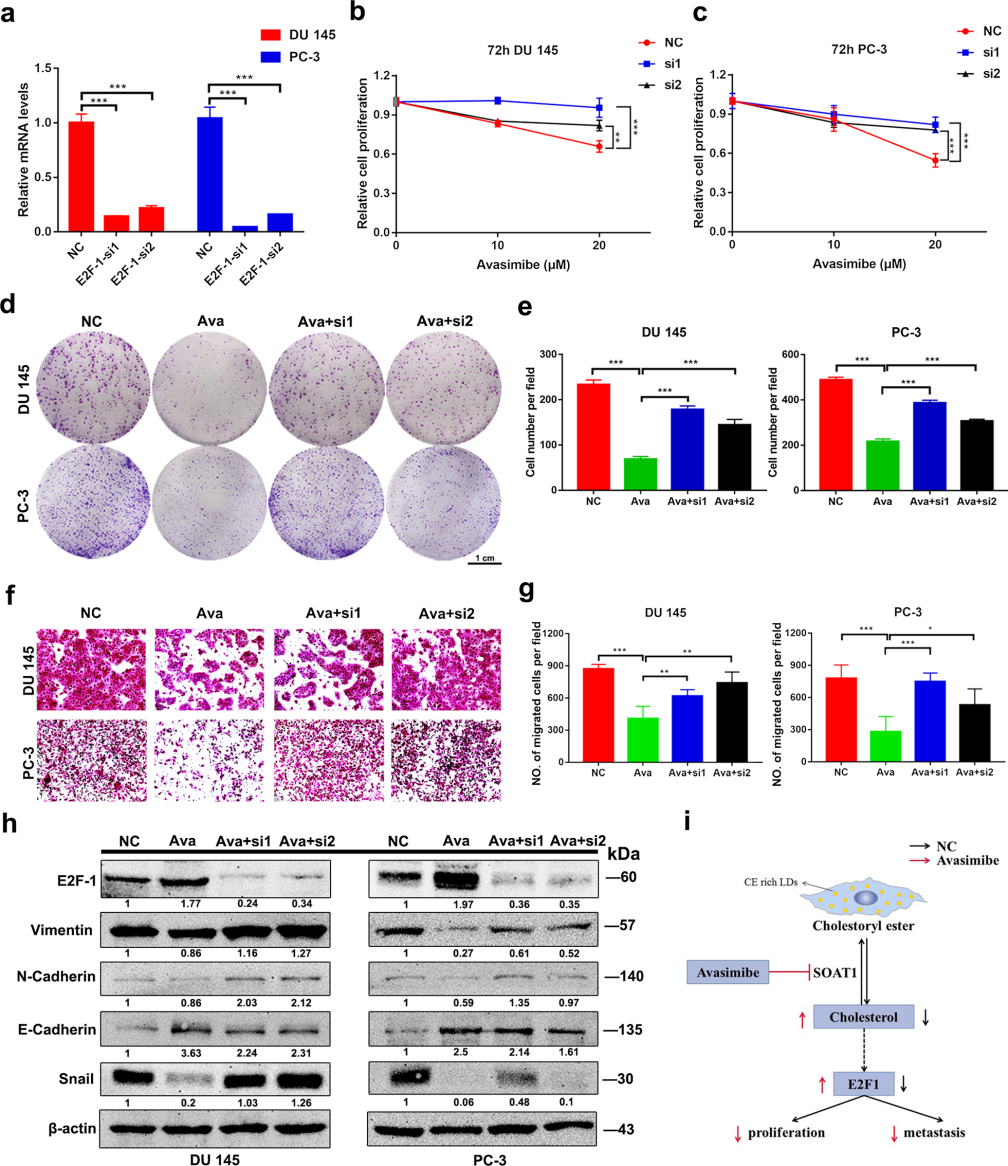
Avasimibe-induced inhibition of cell migration and proliferation was partially rescued by E2F-1 knockdown.** a** qRT-PCR affirmed the interfering efficiency of E2F-1 in PCa cells (DU 145, PC-3); **b** MTT assays showed that inhibition of cell proliferation was partially rescued by E2F-1 knockdown in DU 145 cells; **c** results of MTT assays in PC-3 cells; **d** clonogenic survival assays showed that inhibition of colony formation was partially rescued by E2F-1 knockdown in DU 145 and PC-3 cells; **e** the quantitative results of clonogenic survival assays; **f** Transwell assays revealed that inhibition of cell migration could be rescued by E2F-1 knockdown; **g** statistical analysis of the results of transwell assays; **h** western blots of the EMT-related protein N-cadherin, Vimentin, E-cadherin and Snail in DU 145 and PC-3 cells after avasimibe treatment and E2F-1 knockdown; **i** mechanism diagram. *p < 0.05, **p < 0.01, ***p < 0.001, the scale bar is 1 cm

E2F-1 can maintain a balance of cholesterol and plays a key role in cell proliferation and migration. The E2F-1 protein expression in PCa cells was increased significantly by treatment with avasimibe. After transfection of the two PCa cell lines with E2F-1 siRNA, the knockdown efficiency was confirmed by qRT-PCR (Fig. 5a) and western blots (Fig. 5h). PCa cells were transfected with E2F-1 siRNA for 24 h and then incubated with a high concentration of avasimibe (20 µM). The effect of avasimibe on PCa cell proliferation was determined by MTT assays (Fig. 5b, c) and clonogenic survival assays (Fig. 5d, e). We found that knockdown of E2F-1 could partially rescue the inhibition of cell proliferation caused by avasimibe. The effect of avasimibe on PCa cell migration was assessed by transwell migration assays (Fig. 5f). By transfecting the two PCa cell lines with E2F-1 siRNA, we found that the inhibition of cell migration caused by avasimibe was significantly recovered (Fig. 5g). Moreover, the fraction of cells arrested in G1 was decreased by knockdown of E2F-1 in both PCa cell lines (Additional file 1: Fig. S5a, b). Knockdown of E2F-1 expression promoted proliferation and migration of PCa cells (Additional file 1: Fig. S6).

To further validate this conclusion, we performed western blotting to detect the expression of related proteins. Consistently, avasimibe-induced EMT-related protein alterations were rescued by E2F-1 knockdown, and the expression of the EMT-related proteins Vimentin, N-cadherin, and Snail was increased and that of E-cadherin was decreased in the PCa cells with E2F-1 knockdown and avasimibe treatment compared with the cells treated with avasimibe alone (Fig. 5h). In addition, the expression of the G1 phase-related proteins CDK2/4/6 Cyclin D1 and Cyclin A1 + A2 increased and p21 expression decreased significantly in PCa cells after E2F-1 knockdown and avasimibe treatment (Additional file 1: Fig. S5c).

## Discussion

As one of the most common malignant tumours worldwide, prostate cancer has a high morbidity and mortality, causing a major burden to society [1, 27, 28]. During the last few years, many studies have attempted to find a more effective treatment for CRPC and metastatic prostate cancer [29–31]. Avasimibe seems to be a good option for treating prostate cancer.

As an inhibitor of SOAT, avasimibe has been confirmed to have good biocompatibility and low toxicity in the human body in clinical trials [12, 13]. Previous studies reported that avasimibe can decrease the expression of sterol regulatory element-binding protein by disrupting the balance of cholesterol metabolism to inhibit the proliferation of many kinds of cancer cells [14, 15]. In addition, avasimibe can suppress PCa cell migration by impairing the Wnt/β-catenin pathway [14]. According to recent reports, avasimibe can inhibit the progression of cholangiocarcinoma by targeting the FoxM1-AKR1C1 signalling pathway and induce apoptosis and cycle arrest to inhibit glioma tumour growth [32, 33]. Surprisingly, we found that the cholesterol esterification inhibitor avasimibe can inhibit the expression of SOAT1. Although this has been reported in other cancers, the conclusion is still under debate since the concentration of avasimibe used in the study was cytotoxic [15, 34]. Further studies are needed to explore how avasimibe affects the expression of SOAT1. However, avasimibe does have some drawbacks, and the poor water solubility and short half-life of avasimibe limit its clinical application. Fortunately, recent research has found that these problems can be solved by embedding avasimibe with a fluorescent hyperstar polymer [35]. In our study, we proved that avasimibe can markedly inhibit the proliferation and migration of PCa cells. Moreover, it resulted in an increase in ROS but had no effect on PCa cell apoptosis.

The GEPIA database and GSEA suggest that the SOAT1 protein is highly expressed in prostate cancer samples. In addition, GSEA showed that SOATI protein expression is positively correlated with fatty acid metabolism and adherens junctions, and adherens junctions are closely related to cell migration. Avasimibe suppressed PCa cell metastasis, was verified by wound healing and Transwell migration assays. Moreover, the protein levels of β-catenin, Vimentin, N-cadherin, Snail, and MMP9 were decreased and that of E-cadherin was increased, which are strongly linked to EMT. EMT is a process that promotes the epithelial cells loss of epithelial characteristics and acquire mesenchymal cell phenotypes, which is critical in tumour metastasis and regulated by complex molecular mechanisms [36, 37]. β-catenin, an essential structural component of cell adhesion, can influence the progression of EMT by enhancing transcription to promote cancer progression. Snail, as a zinc-finger transcriptional repressor, controls EMT by suppressing the expression of specific downstream target genes, including the E-cadherin gene [38]. E2F-1 controls transcriptional regulation by activating or suppressing the expression of transcriptional targets, thus regulating EMT-related gene expression. For instance, in oesophageal squamous cell carcinoma, depletion of E2F-1 may accelerated tumour cell migration in vitro by downregulating FAT1 expression [39]. A previous study in PCa cells found that high E2F-1 protein levels significantly inhibited EMT and altered the levels of EMT-related proteins, and this result was consistent with our study [40]. Consequently, we pretreated cells with E2F-1 siRNA and then incubated them with avasimibe, and the inhibition of migration induced by avasimibe in PCa cells was partially rescued. This finding was further supported by the western blot results. To confirm our results, we established a mouse model of pulmonary metastasis, which demonstrated that avasimibe can significantly suppress PCa lung metastasis. This result might indicate that avasimibe could suppress tumour metastasis via the E2F-1 signalling pathway.

Interestingly, in this study, we found that avasimibe could suppress PCa cell proliferation and cause G1 cell cycle arrest. Avasimibe downregulated the levels of the proteins CDK2, CDK4, CDK6, Cyclin D1, and Cyclin A1 + A2 and upregulated the levels of E2F-1 and p21 in PCa cells [41, 42]. p21, as a cyclin kinase inhibitor, contributes to sustained G1 arrest by inhibiting CDK activity [43]. Altogether, we conclude that avasimibe triggers cell cycle arrest by altering the G1-related protein levels. E2F-1, as a master regulator of restriction point and S phase transit, is involved in governing G1/S phase transition [44]. In addition, E2F-1 can downregulate LnRNA RAD51 antisense RNA 1 and Cyclin D1 expression, which causes G1 cell cycle arrest [45, 46]. After knockdown of E2F-1, we observed recovery of the proliferation suppression caused by avasimibe. Cell cycle arrest and the level of proteins involved in the G1 phase can be partly restored by knockdown of E2F-1. Ki67 is used as a proliferation marker to quantify the proliferative activity of tumours. In the xenograft model, avasimibe inhibited tumour growth and reduced the expression of Ki67. These results indicated that avasimibe suppressed tumour growth by upregulating E2F-1 expression (Fig. 5i).

Regarding how avasimibe upregulates E2F-1 protein expression, it has been reported that avasimibe disrupts the balance of cholesterol metabolism in the cell; that is, high levels of free cholesterol increase the E2F-1 protein level [16]. Some research has revealed a close relationship between cholesterol metabolism and E2F-1 protein expression [16], but a direct relationship between E2F-1 and cholesterol has not yet been clearly discovered; this is a significant field of study. All current studies merely reported that the SOAT protein is the drug target of avasimibe. However, we speculate that in addition to affecting cholesterol metabolism, avasimibe may target other proteins, such as AKT. According to some studies, the levels of p-AKT are significantly reduced after treatment with avasimibe [9], and if the levels of p-AKT, the upstream protein of cholesterol metabolism, decrease, cell cycle arrest in the G1 phase will occur.

However, our study had not been verified in patient-derived tissues, which is a major drawback of our article. In addition, these cell phenotypes could not be completely restored by knockdown of E2F-1, and further studies on the molecular mechanisms of avasimibe therapy in PCa are needed. Previous studies have confirmed that daily treatment with avasimibe (15 mg/kg or 75 mg/kg) can effectively inhibit the proliferation and migration of tumours in animal experiments [9, 14]. To alleviate the pain associated with treatment, we reduced the administration frequency by using a concentration of 30 mg/kg avasimibe on alternate days. Further research showed that 15 mg/kg or 75 mg/kg avasimibe did not cause obvious side effects [9, 14], so the study did not include observations of toxicity and side effects, which is a limitation of this study. However, obvious side effects of avasimibe in xenograft models have not been observed, and literature related to the side effects of the drug has not yet been published. Avasimibe, as a systemic antitumour drug, is still in preclinical development, and much work is required to validate its safety and efficacy for clinical applications.

## Conclusions

We found that avasimibe inhibits the proliferation and metastasis of PCa cells in vivo and in vitro. Moreover, avasimibe may attenuate EMT and trigger cell cycle arrest via the E2F-1 signalling pathway in prostate cancer.

## Supplementary Information

**Additional file 1.** Additional Figures and Tables.

## Data Availability

All data generated or analyzed are submitted to the journal.

## Abbreviations

PCa: Prostate cancer
PFA: Paraformaldehyde
GSEA: Gene set enrichment analysis
EMT: Epithelial-mesenchymal transition
LDs: Lipid droplets
CE: Cholesteryl ester
SOAT1: Sterol *O*-acyltransferase1
FBS: Fetal bovine serum
ADTs: Androgen deprivation therapies
H&E: Hematoxylin and eosin
ROS: Reactive oxygen species
GEPIA: Gene Expression Profiling Interactive Analysis
