# Supplementary material for: The cholesterol esterification inhibitor avasimibe suppresses tumour proliferation and metastasis via the E2F-1 signalling pathway in prostate cancer
Source: Cancer Cell Int. 2021 Aug 30;21:461. doi: 10.1186/s12935-021-02175-5 (PMC8407011; doi:10.1186/s12935-021-02175-5)
Supplement: Supplementary file 1 — Additional file 1. Additional Figures and Tables. [file 12935_2021_2175_MOESM1_ESM.docx]

**Additional file**

**Supplementary Figures**

**
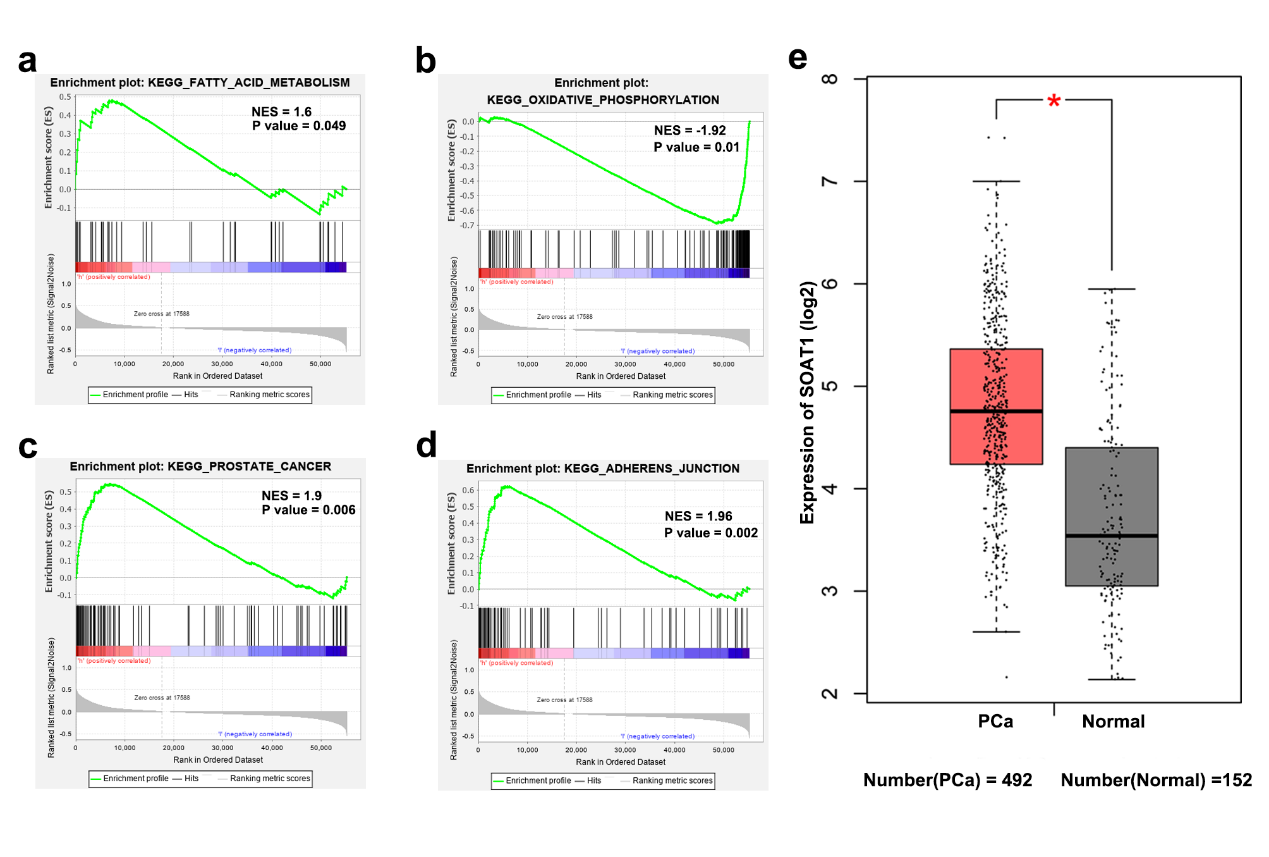
**

**Fig. S****1 GEPIA and GSEA.** **a-d** Four representative functional gene sets enriched (**a**) Fatty acid metabolism; (**b**) oxidative phosphorylation; (**c**) PCa; (**d**) adherens junction. **e** GEPIA database showed SOAT1 was overexpressed in PCa.


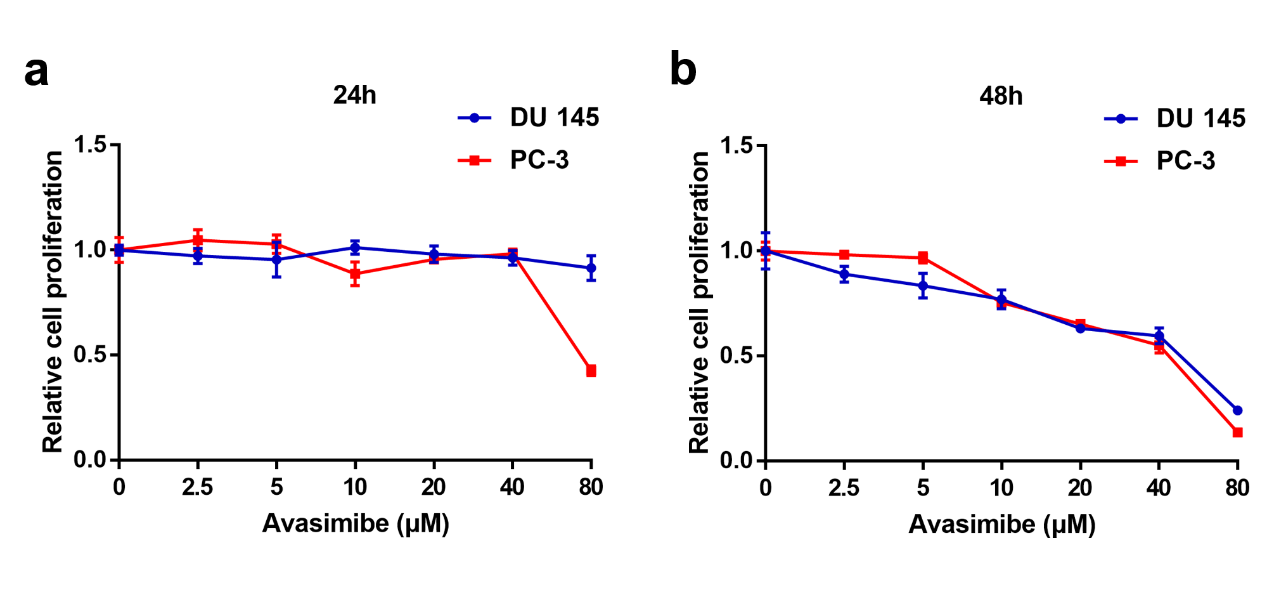


**Fig. S2** **Avasimibe inhibited proliferation in PCa cells. a** MTT assay was used to evaluate the cell growth after treated with avasimibe (0, 0.25, 5, 10, 20, 40 and 80 μM) for 24 h; **b** relative cell proliferation of PCa cells after treated avasimibe for 48 h.


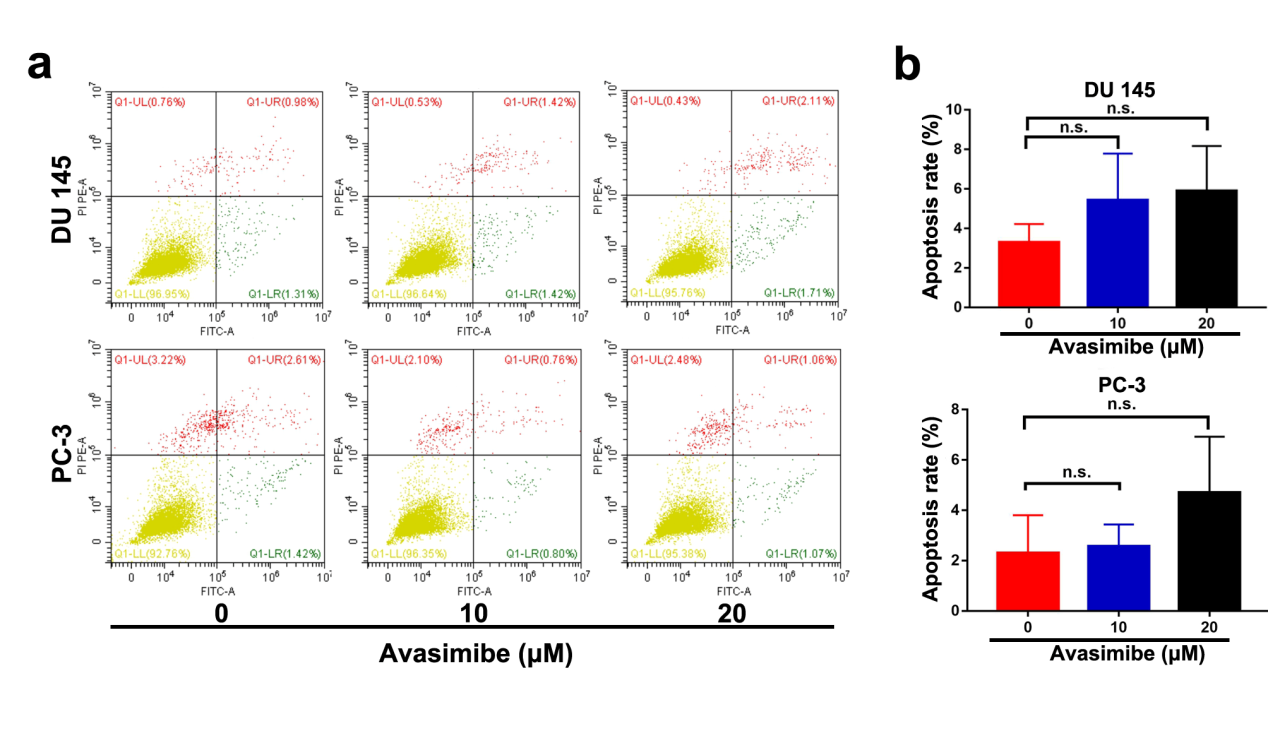


**Fig. S3** **Avasimibe exhibited no effect on apoptosis of PCa cells. a** Flow cytometry analysis for apoptosis rate in PC-3 and DU 145 after treated with avasimibe for 48 h; **b** statistical analysis of apoptosis rate. *n.s*.: no significant differences


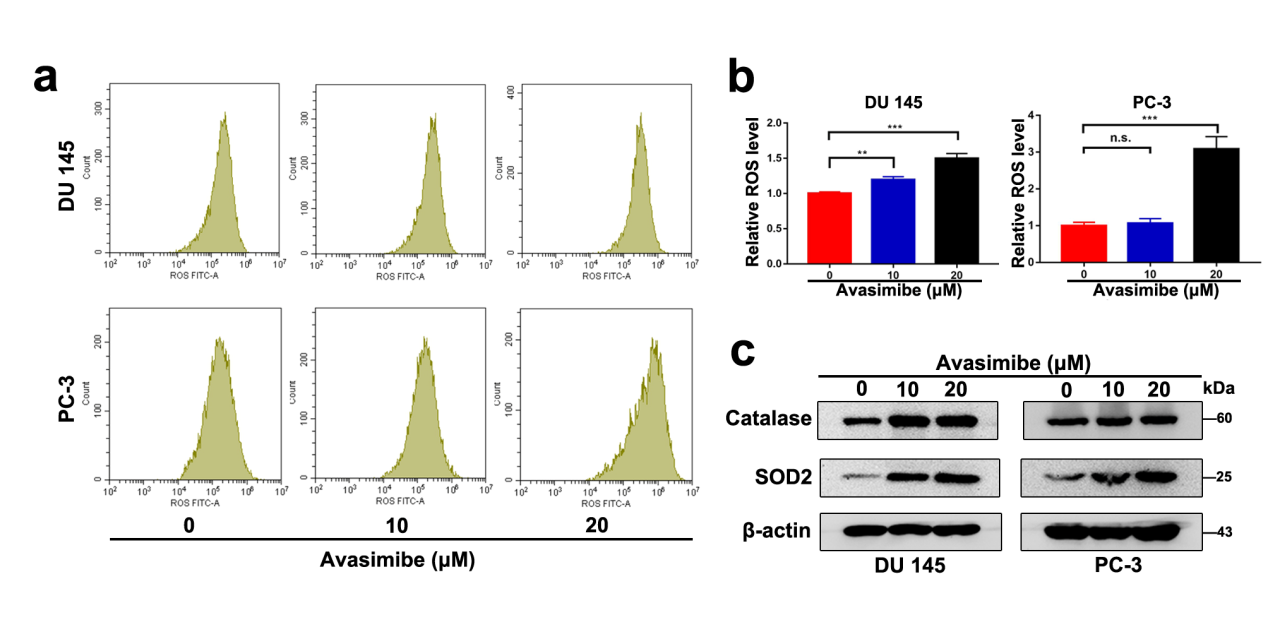


**Fig. S4** **Effect of avasimibe on ROS. a** Flow cytometry analysis of the levels of ROS after treated with avasimibe for 48 h; **b** quantitative results of ROS in DU 145 and PC-3; **c** ROS-related proteins were detected by western blot. *n.s*. no significant differences, **p*<0.05, ***p*<0.01


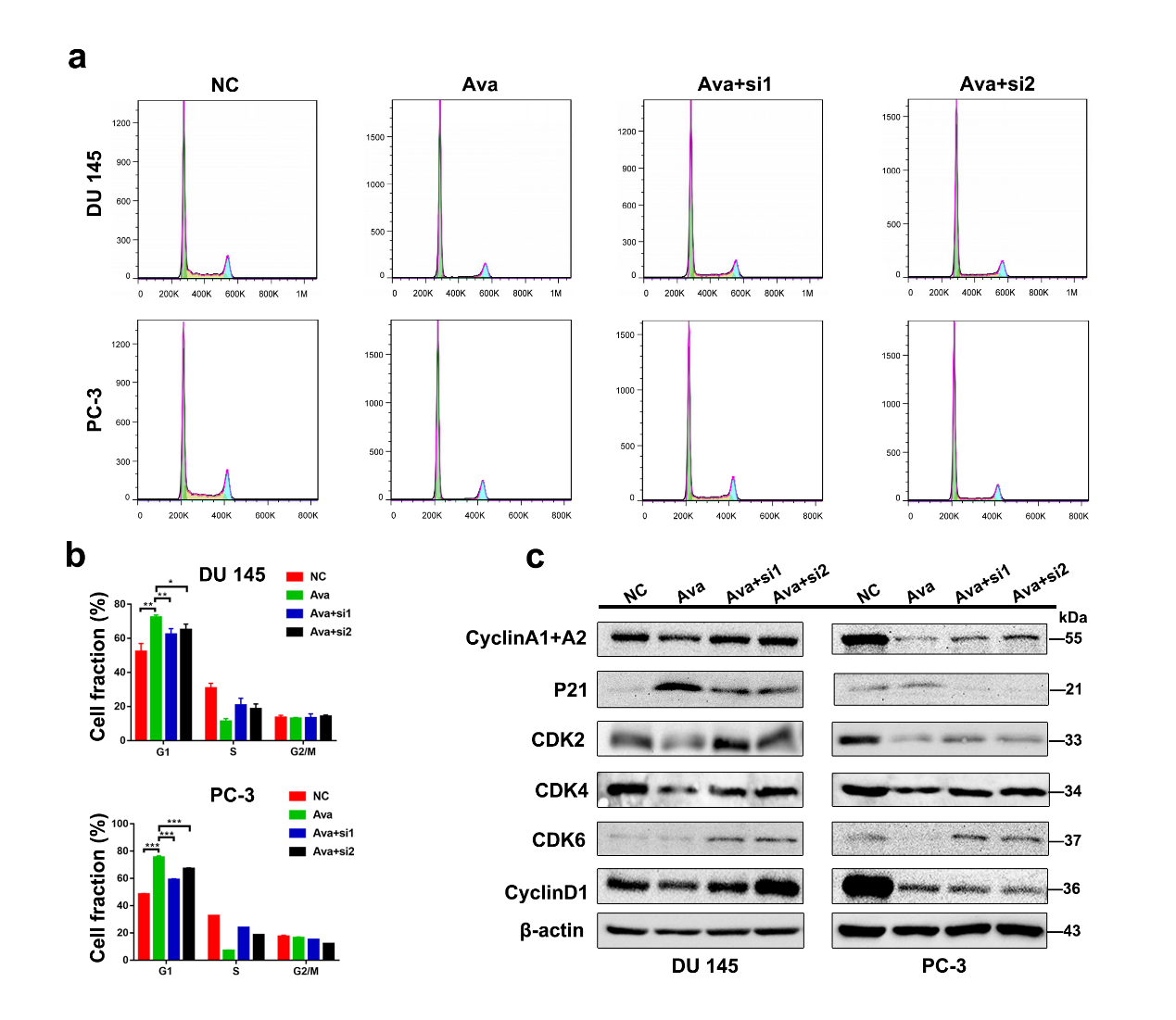


**Fig. S5** **Avasimibe-induced cell cycle arrest was partially rescued by E2F-1 knockdown. a** Flow cytometry analysis showed that arrested G1 cell cycle could be partially rescued by E2F-1 knockdown; **b** statistical analysis of cell fraction in PCa cells; **c** Western blotting of G1-related protein in DU 145 and PC-3 after avasimibe treated and E2F-1 knockdown. **p*<0.05, ***p*<0.01


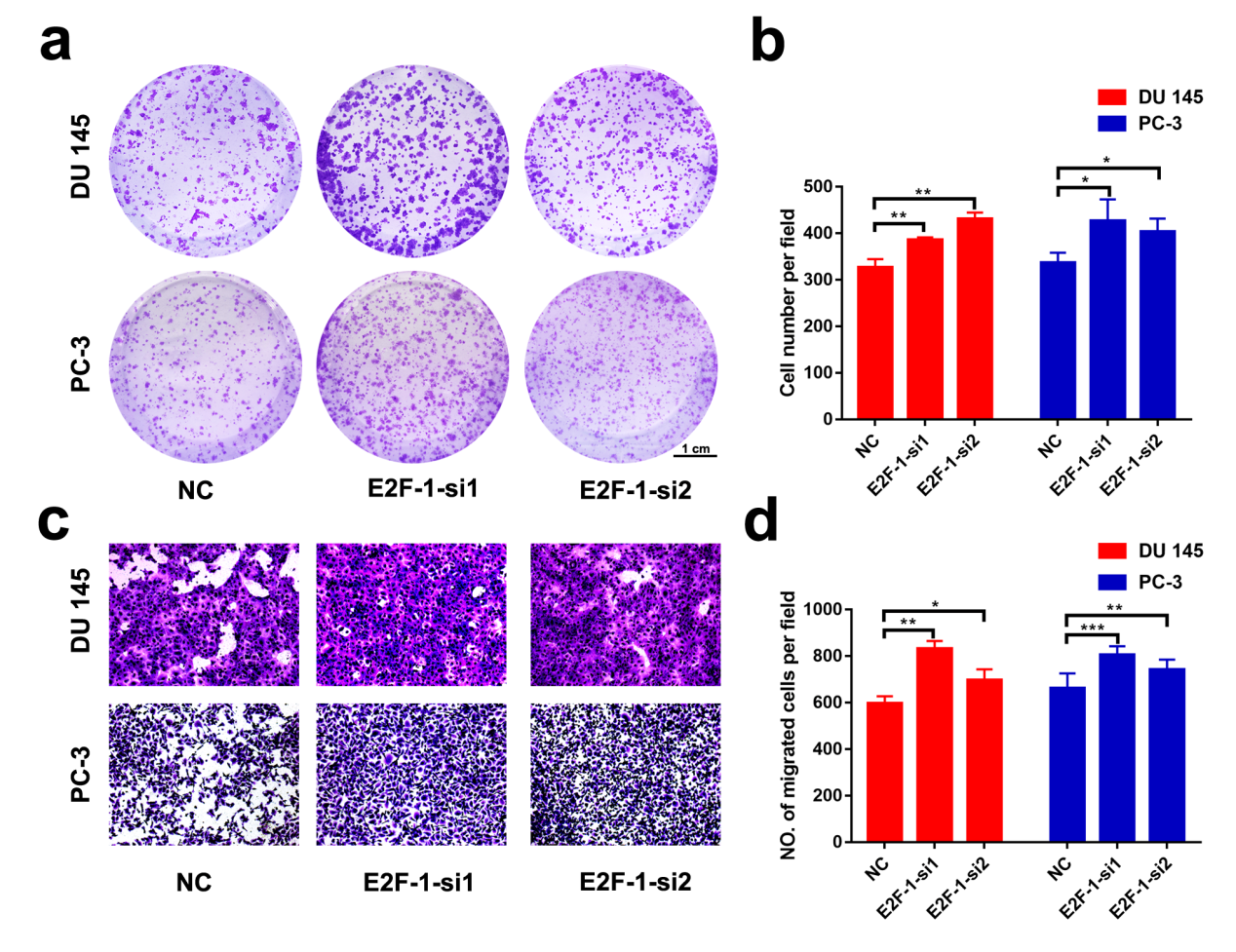


**Fig. S6** **E2F-1 depletion promoted the proliferation and migration of PCa cells.** **a** Clonogenic survival assays of PCa cells after E2F-1 [knockdown](C:/Program%20Files%20(x86)/Youdao/Dict/8.9.6.0/resultui/html/index.html#/javascript:;); **b** statistical analysis of the number of PCa cell colonies formed; **c** Transwell assays of PCa cells after E2F-1 [knockdown](C:/Program%20Files%20(x86)/Youdao/Dict/8.9.6.0/resultui/html/index.html#/javascript:;); **d** quantitative results of the Transwell assays. **p*<0.05, ***p*<0.01, ****p*<0.001

**Supplementary Tables**

**Table S1. List of the essential reagents/chemicals**

| **Reagents/****chemicals** | **Supplier** |
| --- | --- |
| Avasimibe | MedChemExpress, China, Cat. #HY-13215 |
| FBS | Gibco, Australia, Cat. #10099141 |
| RPMI 1640 | Gibco, China, Cat. #C11875500BT |
| DMEM high glucose | HYCLONE, China, Cat. #SH30022.01 |
| Lipofectamine 3000 | Invitrogen，USA, Cat. # L3000075 |
| Opti-MEM® I | Gibco, USA, Cat. #31985-070 |
| iTaq Universal SYBR Green Supermix | Bio-Rad, USA, Cat. #172-5125 |
| HiPure Total RNA Mini Kit | Magen, China, Cat. #R4111-03 |
| Annexin V Cell Apoptpsis Kit | Sungene Biotech, China, Cat. #AO2001-02P-H |
| Cell Cycle Staining Kit | MULTI SCIENCES, China, Cat.#70-CCS012 |
| DCFH-DA | Sigma-Aldrich, China, Cat. #D6883 |
| 4% paraformaldehyde | Biosharp, China, Cat. #BL539A |
| Crystal violet | Biosharp, China, Cat. #BS051 |
| Transwell chamber | Corning, USA, Cat. #353097 |
| ReverTrace qPCR RT Kit | TOYOBO, [Japan](C:/Program%20Files%20(x86)/Youdao/Dict/8.9.6.0/resultui/html/index.html#/javascript:;), Cat. #FSQ-101 |
| Thiazolyl Blue (MTT) | MedChemExpress, China, Cat. #HY-15924 |

**Table S2. List of primers for qRT-PCR.**

| **Symbol** | **Forward primer (5’-3’)** | **Reverse primer (5’-3’)** |
| --- | --- | --- |
| E2F-1 | CATCCCAGGAGGTCACTTCTG | GACAACAGCGGTTCTTGCTC |
| β-actin | CTCGCCTTTGCCGATCC | TTCTCCATGTCGTCCCAGTT |

**Supplementary Table S3. List of primary antibodies.**

| **Antigens** | **Species** | **Dilution (IF)** | **Dilution (WB)** | **Supplier** |
| --- | --- | --- | --- | --- |
| E-cadherin | Rabbit | 1:200 | 1:500 | Cell Signaling Technology, USA, Cat. #3195 |
| N-cadherin | Rabbit | 1:200 | 1:500 | Cell Signaling Technology, USA, Cat. #13116 |
| Vimentin | Rabbit |  | 1:1000 | Cell Signaling Technology, USA, Cat. #5741 |
| Snail | Rabbit |  | 1:500 | Cell Signaling Technology, USA, Cat. #3879S |
| CDK2 | Rabbit |  | 1:1000 | Cell Signaling Technology, USA, Cat. #2546 |
| Cyclin A1+A2 | Rabbit |  | 1:1000 | Abcam, USA, Cat. #ab185619 |
| SOD2 | Rabbit |  | 1:1000 | Abcam, USA, Cat. #ab68155 |
| Catalase | Rabbit |  | 1:1000 | Abcam, USA, Cat. #ab76024 |
| CDK4 | Rabbit |  | 1:1000 | Abcam, USA, Cat. #ab108357 |
| CDK6 | Rabbit |  | 1:1000 | Cell Signaling Technology, USA, Cat. #13331 |
| CyclinD1 | Rabbit |  | 1:1000 | Cell Signaling Technology, USA, Cat. #2978S |
| E2F-1 | Rabbit | 1:200 | 1:500 | ABclonal, China, Cat. #A2067 |
| β-Catenin | Rabbit |  | 1:1000 | Cell Signaling Technology, USA, Cat. #8480T |
| β-actin | Mouse |  | 1:500 | Santa Cruz Biotechnology Inc., USA, Cat. #SC-47778 |
| MMP9 | Rabbit |  | 1:1000 | Cell Signaling Technology, USA, Cat. #13667S |
| P21 | Rabbit |  | 1:1000 | Cell Signaling Technology, USA, Cat. #2947 |
| Ki67 | Rabbit | 1:200 | 1:1000 | Novus Biologicals, China, Cat. #NBP2-19012 |
| SOAT1 | Mouse | 1:200 | 1:1000 | Santa Cruz Biotechnology Inc., USA, Cat. #SC-69836 |

**Supplementary Table S4. List of secondary antibodies and counterstaining of nuclei.**

| **Secondary detection system used** | **Host** | **Method** | **Dilution** | **Supplier** |
| --- | --- | --- | --- | --- |
| Anti-Mouse-IgG (H+L)-HRP | Goat | WB | 1:10,000 | Jackson ImmunoResearch Inc, USA, Cat. #115-005-003 |
| Anti-Rabbit-IgG (H+L)-HRP | Goat | WB | 1:5,000 | Jackson ImmunoResearch Inc, USA, Cat. #111-005-003 |
| Goat anti-Rabbit IgG (H+L) Highly Cross-Adsorbed Secondary Antibody, Alexa Fluor 594 | Goat | IF | 1:1,000 | Invitrogen, USA, Cat. #A-11037 |
| Goat anti-Mouse IgG (H+L) Highly Cross-Adsorbed Secondary Antibody, Alexa Fluor 488 | Goat | IF | 1:1,000 | Invitrogen, USA, Cat. #A-11029 |
| DAPI | - | IF | 1:1,000 | Invitrogen, USA, Cat. #D1306 |
